# Carcinoembryonic antigen and cytokeratin-19 fragments for assessment of therapy response in non-small cell lung cancer: a systematic review and meta-analysis

**DOI:** 10.1038/bjc.2017.45

**Published:** 2017-03-09

**Authors:** Stefan Holdenrieder, Birgit Wehnl, Karina Hettwer, Kirsten Simon, Steffen Uhlig, Farshid Dayyani

**Affiliations:** 1 Institute of Clinical Chemistry and Clinical Pharmacology, University Hospital Bonn, Sigmund-Freud-Str. 25, Bonn D-53105, Germany; 2 Roche Diagnostics GmbH, Nonnenwald 2, Penzberg 82377, Germany; 3 QuoData GmbH, Prellerstrasse 14, Dresden 01309, Germany; 4 Division of Hematology/Oncology, Department of Medicine, University of California Irvine, 101 The City Drive South, Orange, CA 92868, USA

**Keywords:** CEA, CYFRA 21-1, prediction, monitoring, meta-analysis, response

## Abstract

**Background:**

This meta-analysis evaluated whether pretherapy serum levels of carcinoembryonic antigen (CEA) and cytokeratin-19 fragments (CYFRA 21-1) are predictive of response to therapy in non-small cell lung cancer (NSCLC) and whether changes in these markers during *vs* pretherapy are indicative of response.

**Methods:**

Original peer-reviewed studies enrolling adults with untreated advanced NSCLC were identified using PubMed. Two reviewers independently extracted data from eligible studies and assessed study heterogeneity and the risk of study bias.

**Results:**

Fourteen studies were eligible; 11 had objective response as an end point and three evaluated clinical benefit (i.e., response and stable disease). Study bias was relatively low. Both markers showed comparable modest predictive value across studies, with baseline CYFRA 21-1 numerically better in predicting treatment benefit. A good performance in identifying objective response during treatment was seen (AUC 0.724 (95% CI 0.667–0.785) for CYFRA 21-1 and 0.728 (95% CI, 0.599–0.871) for CEA). A decline in CYFRA 21-1 levels during treatment was highly indicative for objective response (sensitivity 79.1% (95% CI 71.5–85.1)).

**Conclusions:**

Comprehensive analysis of study heterogeneity and bias provides a high level of evidence for the clinical utility of CEA and CYFRA 21-1 for the prediction and monitoring of response in NSCLC.

Most patients with non-small cell lung cancer (NSCLC) present with advanced disease at diagnosis. In this setting, therapeutic options are limited to systemic treatments, including targeted agents and increasingly immunotherapy, and in select cases radiotherapy. However, response to treatment is heterogeneous (Gridelli *et al*, 2003; National Comprehensive Cancer Network, 2016). As therapy is associated with significant adverse events, it is imperative to ensure that patients are actually benefitting from treatment. Early identification of progressive disease (PD) during treatment is vital to save time and costs in switching to a new treatment strategy and to avoid unnecessary side effects from exposure to an ineffective regimen (Holdenrieder *et al*, 2008; Holdenrieder and Stieber, 2010).

Imaging techniques are routinely used in NSCLC to monitor response to chemotherapy, but these are associated with relatively high costs and are inconvenient for patients (Mahadevia *et al*, 2003; Ganti and Mulshine, 2006; National Comprehensive Cancer Network, 2016). A number of established biomarkers have been utilised in NSCLC for diagnosis, prognosis and therapeutic monitoring (Barak *et al*, 2010; Holdenrieder *et al*, 2010; Molina *et al*, 2010). Carcinoembryonic antigen (CEA) and cytokeratin-19 fragments (CYFRA 21-1) are widely used in certain regions of the world, such as Eastern Asia (Zhi *et al*, 2015), for differential diagnosis in NSCLC (Schalhorn *et al*, 2001; Molina *et al*, 2003, 2010, 2016) and have demonstrated great potential for predicting early response to chemotherapy (Vollmer *et al*, 2003; Holdenrieder *et al*, 2004, 2006).

We conducted a systematic review and meta-analysis to evaluate CEA and CYFRA 21-1 in the assessment of therapy response in NSCLC. Specifically, we aimed to address two clinical questions. First, are pretherapy serum levels of CEA and CYFRA 21-1 predictive of response to therapy in patients with previously untreated advanced NSCLC? Second, are changes in serum marker levels of CEA and CYFRA 21-1 during therapy, as compared with pretherapy levels, indicative of response in patients with previously untreated advanced NSCLC?

## Materials and methods

### Literature search

We searched PubMed for studies published between 1 January 2000 and 23 June 2015 using the terms: CEA (cea, carcinoembryonic, carcino-embryonic, carcino embryonic), CYFRA 21-1 (cyfra, cytokeratin 19, cytokeratin-19), and NSCLC (nsclc, non-small cell lung cancer, non-small cell lung carcinoma, lung adenocarcinoma, lung squamous cell carcinoma, adenocarcinoma of the lung, squamous cell carcinoma of the lung, lung cancer, lung carcinoma). Search terms relating to the assessment of therapy response were not included owing to the inconsistent use of terms describing tumour markers in the literature. The meta-analysis was registered with PROSPERO (registration no. CRD42015029974) (Uhlig *et al*, 2015).

### Eligibility criteria

All original peer-reviewed research publications were considered, including prospective and retrospective studies. Eligible studies were required to: enrol adults with advanced NSCLC receiving first-line therapy; classify patients as ‘high' or ‘low/normal' with respect to CEA and CYFRA 21-1 serum levels prior to therapy and/or classify patients as having ‘reduction' or ‘no reduction' according to changes in marker levels during treatment; classify patients as ‘responder' or ‘non-responder' to therapy (based on World Health Organization (WHO)/Response Evaluation Criteria in Solid Tumors (RECIST) criteria); and determine serum marker levels using commercially available assays.

Review articles, systematic reviews, meta-analyses, conference abstracts and case studies were excluded, as were preclinical studies. Non-English language studies were excluded during screening.

### Data extraction

Two reviewers independently extracted the following data from each study: bibliographic information; study methodology; number of patients, demographics and baseline characteristics; patient numbers for subgroups; disease and treatment characteristics; assay characteristics; marker-level characteristics; assessment of therapy with respect to objective tumour response; and statistical measures for therapy response with confidence intervals (CIs) if available. No numerical information was extracted from the figures in the study publications.

### Statistical methods

Statistical analyses were performed using R programming software (The R Foundation, 2015).

#### Risk of bias

The risk of bias in individual studies and across studies (publication bias) was analysed independently by two reviewers and evaluated by means of Begg's funnel plot and Egger's test for asymmetry, with *P*⩽0.05 considered significant (Egger *et al*, 1997). The risk of bias in individual studies (i.e., from patient selection, biomarker tests, reference standards, flow and timing or results of statistical analysis) was assessed based on the Quadas-2 checklist, modified and extended as appropriate to the question of therapy response assessment (Whiting *et al*, 2011). As not only publication bias but also study bias could produce outliers or asymmetry in funnel plots, results of the formal statistical tests on asymmetry should be interpreted with caution.

#### Meta-analysis and study heterogeneity

Three statistical measures were considered: area under the curve (AUC) (Hanley and McNeil, 1982; Walter, 2002); sensitivity and specificity (Rutter and Gatsonis, 2001; Reitsma *et al*, 2005); and diagnostic odds ratio (DOR) (Glas *et al*, 2003). The AUC and the DOR are univariate approaches to quantifying the quality of a diagnostic test. In the present meta-analysis, the diagnostic test was the correct classification of patients as responders or non-responders to therapy by means of the tumour marker CEA or CYFRA 21-1. The AUC is the integral of the Receiver Operating Characteristic (ROC) curve. An AUC of 1 represents a perfect test, while an AUC of 0.5 represents a test that does not discriminate between two groups. The sensitivity of a diagnostic test refers to the ability of the test to correctly identify a certain group of patients. Sensitivity and specificity values range from 0 to 1 and should be as high as possible. The DOR can take on any value >0. For useful diagnostic tests, the DOR is >1; higher DOR values are indicative of higher discriminatory power.

#### Area under the curve

AUC analysis was carried out using extracted AUC values and corresponding standard errors. A summary AUC was computed on the basis of a linear model with a random effect for the study bias. The linear model was set up on the basis of the logit-transformed AUC values: ln *P/*1−*P*, with *P*=2·AUC −1.

For each AUC result, Tau squared (*T*^2^), *Q* and *I* squared (*I*^2^) were calculated. *T*^2^ denotes the variance of the random variable representing study bias. *Q* was calculated as the weighted sum of squared differences between individual study effects and the pooled effect across studies; it is distributed as a chi-square statistic with *k*−1 degrees of freedom (*k* is the number of studies). *I*^2^ represents the percentage of the variability due to heterogeneity between studies rather than to chance; a value >50% is considered heterogeneous.

#### Sensitivity and specificity

Sensitivity and specificity analyses were performed using contingency tables provided in the studies or, if missing, computed from extracted data. By means of a bivariate model, estimates for the logit of both sensitivity and specificity values were calculated. It was assumed that these two parameter estimates followed a bivariate normal distribution with a covariance matrix whose entries were also estimated. The study bias was modelled as a random effect. *T*^2^ values were calculated for each sensitivity and specificity result.

#### Diagnostics odds ratio

DOR analysis was carried out on the basis of contingency tables. For the studies by Wang *et al* (2010) and Wang *et al* (2011), sensitivity and specificity values for several cutoffs were reported; DORs were calculated for the different cutoff values and the result with the highest DOR was chosen for further analysis. A summary logarithmic (ln) DOR was computed on the basis of a linear model with a random effect for the study bias. *T*^2^ values were calculated for each ln DOR result.

### Meta-regression

A meta-regression was performed to assess the effect of ethnic group (Asian *vs* non-Asian), assay (manual *vs* automated) and NSCLC stage (III–IV *vs* I–IV) on the ln DOR for response.

## Results

### Study selection

**Figure 1:**
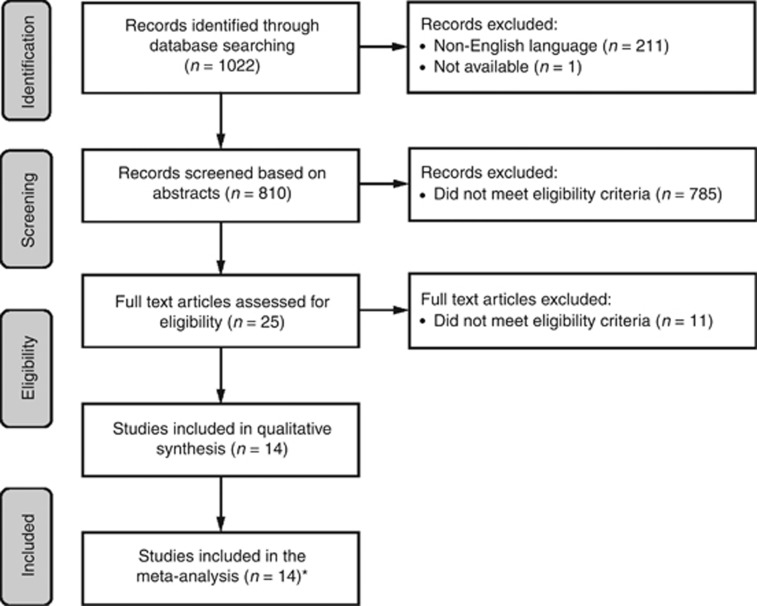
PRISMA flow diagram of eligible studies.*Initially, only three studies reported results for patients with advanced (stages IIIB or IV) NSCLC. The decision was therefore made to include all NSCLC stages in the meta-analysis, meaning that 14 studies were eligible for inclusion. Of these, 11 had objective response (complete or partial response) as an end point and the other three evaluated CEA and CYFRA 21-1 for their ability to show clinical benefit (i.e., response and stable disease during treatment).

Of the 1022 records identified, 25 studies were deemed eligible based on abstract screening (Figure 1). Eleven of these were subsequently excluded as they did not fulfil the inclusion criteria (Supplementary Table S1). Only three of the remaining studies reported results for advanced (stages IIIB or IV) NSCLC. The decision was therefore made to include all NSCLC stages in the meta-analysis, meaning that all of the 14 remaining studies were eligible (Supplementary Table S2).

Just three studies focussed on the identification of patients with PD *vs* those with clinical benefit (i.e., response or stable disease (SD)) (Holdenrieder *et al*, 2006; Nisman *et al*, 2008; Arrieta *et al*, 2013). As this distinction is of clinical relevance in the setting of treatment monitoring, data on the identification of patients with PD were extracted from a further three studies (Trapé *et al*, 2003; Jin *et al*, 2010; Wang *et al*, 2010). Most of the studies focussed on the clinical response end point (complete response (CR) or partial response (PR)) rather than the clinical benefit (which includes SD) and monitoring end point.

### Study classification

The 14 studies were classified in terms of response definition, tumour markers assessed and whether these were evaluated as predictive or treatment monitoring markers. Eleven studies defined response as CR plus PR and compared the diagnostic performance of CEA and CYFRA 21-1 to distinguish CR+PR from SD+PD, defined as ‘non-responders'. The other classification evaluated CEA and CYFRA 21-1 as markers for clinical benefit, that is, CR+PR+SD *vs* patients with PD.

To reflect the change in eligibility criteria, the two clinical questions being addressed by the meta-analysis were modified to refer to all stages of NSCLC rather than just patients with advanced disease and are referred to as ‘Prediction' and ‘Treatment monitoring' throughout the manuscript.

An overview of the 14 eligible studies is provided in Table 1 for the comparison (CR+PR) *vs* (SD+PD) and in Table 2 for the comparison (PD) *vs* (CR+PR+SD). For the comparison (CR+PR) *vs* (SD+PD), four studies analysed both markers for each clinical question and two studies analysed both clinical questions for each marker (Supplementary Table S3).

### Publication bias

For the ‘Treatment monitoring' question and the comparison (CR+PR) *vs* (SD+PD), the study by Arrieta *et al* (2013) fell outside the 99% CI for CEA (AUC 0.83, Egger's test *P*=0.038; Supplementary Figure S1). More detailed analysis of this study indicated that there was a study bias, rather than a publication bias, possibly because only patients with baseline serum CEA>10 ng ml^−1^ were included. For CYFRA 21-1, all studies were within the 99% CI and there was no evidence of publication bias (AUC 0.72, Egger's test *P*=0.847).

Supplementary Figure S2 shows bias analysis using funnel plots for DOR for the comparison (CR+PR) *vs* (SD+PD). With one exception, all of the studies were within the 99% CI. For the ‘Treatment monitoring' question and the comparison (CR+PR) *vs* (SD+PD), the study by Arrieta *et al* (2013) was again outside the 99% CI for CEA; however, no publication bias was present (DOR 5.0, Egger's test *P*=0.153), hence it was not necessary to exclude this study from the meta-analysis. No bias was detected for DOR for the comparison (PD) *vs* (CR+PR+SD) for either of the clinical questions or tumour markers (Egger's test *P*⩾0.05).

### AUC results

Only for the comparison (CR+PR) *vs* (SD+PD) and the ‘Treatment monitoring' question were enough studies available for the meta-analysis. Two meta-analyses were performed for AUC with and without the study by Arrieta *et al* (2013), but no significant difference was found between them (Grubbs outlier test at the 1% significance level: AUC 0.728 (95% CI 0.599–0.871) and 0.667 (95% CI 0.606–0.742)), respectively). Removing the study reduced the level of heterogeneity from 88.4% to 38.7%.

**Figure 2:**
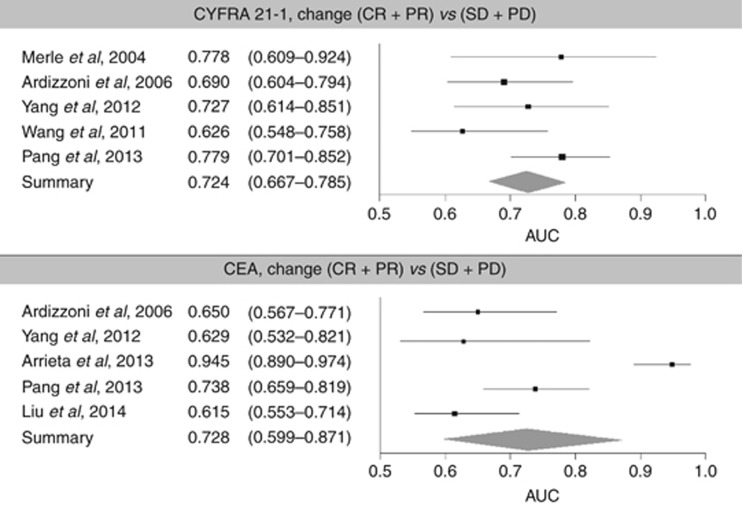
Forest plots for AUC for the comparison (CR+PR) *vs* (SD+PD).

Across-study AUC values for all combinations of response comparison, marker and clinical question were similar for the two markers, with a summary AUC of 0.728 (95% CI 0.599–0.871) for CEA and 0.724 (95% CI 0.667–0.785) for CYFRA 21-1 (Table 3; Figure 2), indicating good predictive power and clinical significance. As the CIs for the two markers overlapped, the predictive power of CEA and CYFRA 21-1 was comparable.

### Sensitivity/specificity results

Contingency tables for the two clinical questions are shown in Supplementary Table S4.

**Figure 3:**
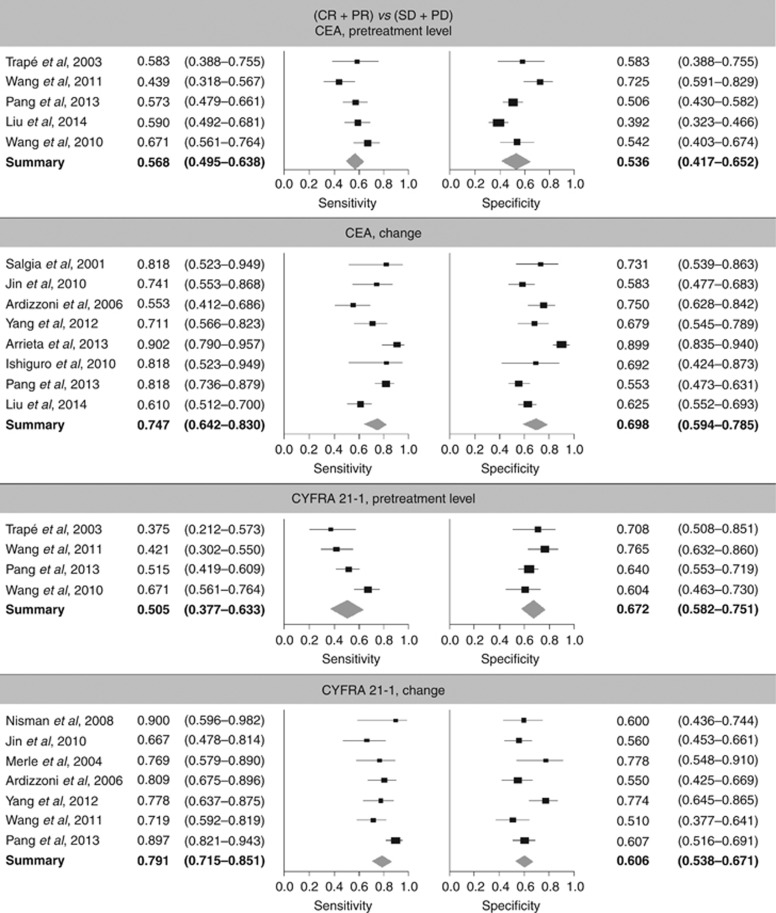
Forest plots for sensitivity and specificity for the comparison (CR+PR) *vs* (SD+PD).

In the assessment of the predictive performance for subsequent (CR+PR) *vs* (SD+PD), across-study sensitivity for response with CEA was 56.8% (i.e., 56.8% of patients with response had a pretreatment level below the cutoff value) and specificity was 53.6% (i.e., 53.6% of patients with SD or PD had a pretreatment level above the cutoff value) (Table 3; Figure 3). Corresponding values for CYFRA 21-1 were 50.5% and 67.2%, respectively. The CIs of sensitivity and specificity for both markers overlapped, indicating comparable predictive power.

To assess the performance of the markers to indicate (CR+PR) *vs* (SD+PD) during treatment, meta-analyses for sensitivity and specificity for response were performed with and without the study by Arrieta *et al* (2013). Sensitivity for response was 74.7% with CEA (i.e., 74.7% of patients with CR/PR had a ‘strong' reduction in marker level) and specificity was 69.8% (i.e., 69.8% of patients with SD/PD had a ‘weak' reduction in marker level). For CYFRA 21-1, sensitivity and specificity for response were 79.1% and 60.6%, respectively (Table 3; Figure 3). No significant differences between CEA and CYFRA 21-1 were observed. Study-specific cutoffs and sensitivity and specificity values for the comparison (CR+PR) *vs* (SD+PD) are shown in Supplementary Figure S3.

For the comparison (PD) *vs* (CR+PR+SD), the CIs of sensitivity and specificity for progression for both markers overlapped for both clinical questions, indicating comparable predictive power (Table 3).

### DOR results

Across-study DOR and ln DOR values are summarised in Supplementary Table S5, with corresponding forest plots shown in Supplementary Figure S4.

The DOR for response was defined as the ratio of two odds:

DOR_response_=odds that response will occur, given a low marker level/odds that response will occur, given a high marker level.

For the comparison (CR+PR) *vs* (SD+PD) and the ‘Prediction' question, the DOR for response was 1.49 (95% CI 1.03–2.16) with CEA and 2.16 (95% CI 1.49–3.13) with CYFRA 21-1 (Supplementary Table S5).

For the assessment of the predictive performance for subsequent (CR+PR) *vs* (SD+PD), meta-analyses for DOR for response were also performed with and without the study by Arrieta *et al* (2013). The DOR for response with CEA was 6.89 (95% CI 3.40–13.95) compared with 6.42 (95% CI 3.50–11.79) with CYFRA 21-1. The CIs were significantly different from 1 for both markers for both clinical questions, showing evidence of their clinical relevance as predictors of treatment response. Between-study heterogeneity for all combinations of marker and clinical question was very low (Supplementary Table S5).

The DOR for progression was defined as the ratio of two odds:

DOR_progression_=odds that progression will occur, given a high marker level/odds that progression will occur, given a low marker level.

Assessment of the predictive performance for subsequent (PD) *vs* (CR+PR+SD) showed the DOR for progression was 1.82 (95% CI 0.97–3.41) with CEA and 3.16 (95% CI 2.01–4.96) with CYFRA 21-1 (Supplementary Table S5). To assess the performance of the markers to indicate PD during treatment, the DOR for progression was 1.97 (95% CI 0.48–8.09) with CEA *vs* 14.73 (95% CI 5.01–43.29) with CYFRA 21-1, demonstrating the clinical significance of CYFRA 21-1 for the evaluation of treatment response.

### Meta-regression analysis

The feasibility of subgroup analysis was assessed for all combinations of response comparison, marker and clinical question and the statistical measures AUC, sensitivity/specificity and DOR (Supplementary Table S6; Supplementary Table S7). No significant differences between ethnic groups, assay type or tumour stage were detectable.

## Discussion

This systematic review and meta-analysis aimed to determine whether pretreatment serum levels of CEA and CYFRA 21-1 are predictive of response to therapy in previously untreated NSCLC (‘Prediction'), and whether changes in serum levels during therapy are indicative of response in this patient population (‘Treatment monitoring').

For the comparison (CR+PR) *vs* (SD+PD), AUC data indicated good predictive power for CYFRA 21-1. However, for CEA, a high level of heterogeneity was observed as a result of inclusion of the study by Arrieta *et al* (2013). There were too few studies for a meaningful computation of summary ROC curves.

Across-study sensitivity and specificity results were better for ‘Treatment monitoring' than for ‘Prediction', indicating that the change in CEA and CYFRA 21-1 level had a higher predictive power than the pretreatment level alone. For both clinical questions, results for CYFRA 21-1 were superior to those for CEA. However, owing to the limited number of studies, and the resulting large CIs, no clear conclusion as to the clinical significance of the two markers for the comparison (PD) *vs* (CR+PR+SD) could be drawn.

Across-study DOR results for the comparison (CR+PR) *vs* (SD+PD) for both markers were superior for ‘Treatment monitoring' compared with ‘Prediction', again indicating the higher predictive power of the change in CEA and CYFRA 21-1 levels compared with the pretreatment level alone. For all four combinations of marker and clinical question, the DOR for response was significantly >1, supporting the clinical utility of the two markers in this setting. The DOR for response values for the ‘Treatment monitoring' question for both markers also provided evidence of high discriminatory power. There were no significant differences in DOR for response between ethnic groups, assay type or tumour stage, lending further validity to the overall results.

For DOR and the comparison (PD) *vs* (CR+PR+SD), the CI for the DOR for progression for CEA included 1 for both prediction and monitoring use, indicating that neither pretreatment level nor change in level correlated with response. However, the results for CYFRA 21-1 showed the usefulness of the marker for prediction and therapy monitoring. As very few studies were available for the comparison (PD) *vs* (CR+PR+SD), a reliable conclusion as to the clinical significance of the markers with respect to this comparison was not possible. However, by calculating systematic bias of single studies in this meta-analysis, we obtained a much higher level of evidence for the performance of CEA and CYFRA 21-1 as biomarkers.

A number of potential sources of between-study heterogeneity and uncertainty in the meta-analysis should be considered. The use of different tumour response classifications may have resulted in varying numbers of responders and non-responders. The cutoff values chosen to represent the discriminatory power of the markers in the different studies were consistent for the ‘Prediction' question but varied considerably for the ‘Treatment monitoring' question. Patients with different stages of NSCLC were included, which may have confounded the predictive power of the markers. In addition, the studies used different patient selection criteria. This was particularly notable for the study by Arrieta *et al* (2013), which only enrolled patients with high serum CEA levels at baseline, although outlier tests proved negative and CIs for the results with and without the study by Arrieta *et al* (2013) overlapped. Finally, while great care was taken to ensure homogeneity of the time points for the evaluation of tumour response and marker measurements across the studies, some differences would have been inevitable (but in most studies, venipunctures were performed at the time of imaging investigations for staging).

In conclusion, results of this meta-analysis demonstrate the clinical utility of both CEA and CYFRA 21-1 for the assessment of response to therapy in NSCLC. The performance for both markers was stronger for treatment monitoring than for predictive value at baseline. With respect to the question of detecting progression during treatment (the reason, for example, why interim imaging is carried out in between chemotherapy cycles), results for CYFRA 21-1 suggested high discriminatory power, though a larger number of studies would have been preferable.

The results of this comprehensive analysis are highly relevant for the clinical management of lung cancer patients, as a majority do not yet benefit from new targeted therapy approaches. The development of well-defined criteria for the use of established cancer biomarkers will be essential as a complementary strategy for the sensitive guidance of these patients.

## Figures and Tables

**Table 1:** Summary of available studies for the comparison (CR+PR) *vs* (SD+PD)

**Marker**	**Clinical question**	**Studies,** ***n***	**Study**	**Stages**	**Second marker measurement**^a^	**Responders/non-responders,** ***n***	**SN/SP available**	**AUC available**
			Trapé *et al*, 2003	IIIA, IIIB, IV	—	24/24	Yes	No
			Wang *et al*, 2011	I, II, III, IV	—	57/51	Yes	No
	Prediction	5	Pang *et al*, 2013	I, II, IIIA, IIIB, IV	—	110/162	Yes	No
			Liu *et al*, 2014	I, II, III, IV	—	100/176	Yes	No
			Wang *et al*, 2010	I, II, III, IV	—	79/48	Yes	Yes
			Salgia *et al*, 2001	III/IIIB, IV^b^	2 months after chemo	11/26	Yes	No
			Jin *et al*, 2010	IIIB, IV	After second cycle	27/84	Yes	No
CEA			Ardizzoni *et al*, 2006	III, IV	After second cycle	47/60	Yes	Yes
			Yang *et al*, 2012	IIIB, IV	After second cycle	45/53	Yes	Yes
	Treatment	8	Arrieta *et al*, 2013	III, IV	After second cycle	51/129	Yes	Yes
	monitoring		Ishiguro *et al*, 2010	IB, IIB, IIIA, IIIB	After chemo, before surgery	11/13	Yes	No
			Pang *et al*, 2013	I, II, IIIA, IIIB, IV	3 weeks after second/fourth cycle	110/150	Yes	Yes
			Liu *et al*, 2014	I, II, III, IV	After second cycle	100/176	Yes	Yes
			Trapé *et al*, 2003	IIIA, IIIB, IV	—	24/24	Yes	No
			Wang *et al*, 2011	I, II, III, IV	—	57/51	Yes	No
	Prediction	4	Pang *et al*, 2013	I, II, IIIA, IIIB, IV	—	103/125	Yes	No
			Wang *et al*, 2010	I, II, III, IV	—	79/48	Yes	Yes
			Nisman *et al*, 2008	IIIA, IIIB, IV	After second cycle	10/35	Yes	No
CYFRA 21-1			Jin *et al*, 2010	IIIB, IV	After second cycle	27/84	Yes	No
			Merle *et al*, 2004	IIIA, IIIB	After first cycle	26/18	Yes	Yes
	Treatment	7	Ardizzoni *et al*, 2006	III, IV	After second cycle	47/60	Yes	Yes
	monitoring		Yang *et al*, 2012	IIIB, IV	After second cycle	45/53	Yes	Yes
			Wang *et al*, 2011	I, II, III, IV	After second cycle	57/51	Yes	Yes
			Pang *et al*, 2013	I, II, IIIA, IIIB, IV	3 weeks after second/fourth cycle	97/117	Yes	Yes

					**Patients with corresponding stage,** ***n***^c^									
**Marker**	**Clinical question**	**Studies,** ***n***	**Study**	**Stages**	**I**	**IB**	**II**	**IIB**	**III**	**IIIA**	**IIIB**	**IV**	**Unknown**	**Patients with stage III–IV, %**
			Trapé *et al*, 2003	IIIA, IIIB, IV						10	17	21		100
			Wang *et al*, 2011	I, II, III, IV	1		5		64			38		94.4
	Prediction	5	Pang *et al*, 2013	I, II, IIIA, IIIB, IV	34		44			27	28	143		71.7
			Liu *et al*, 2014	I, II, III, IV	39		43		137			401	69	86.8
			Wang *et al*, 2010	I, II, III, IV	5		14		66			42		85.0
			Salgia *et al*, 2001	III/IIIB, IV^b^	(68)		(23)		46			79		100^d^
CEA			Jin *et al*, 2010	IIIB, IV							39	72		100
			Ardizzoni *et al*, 2006	III, IV					41			76		100
	Treatment	8	Yang *et al*, 2012	IIIB, IV					53			45		100
	monitoring		Arrieta *et al*, 2013	III, IV					28			152		100
			Ishiguro *et al*, 2010	IB, IIB, IIIA, IIIB		1		5		15	3			75.0
			Pang *et al*, 2013	I, II, IIIA, IIIB, IV	34		44			27	28	143		71.7
			Liu *et al*, 2014	I, II, III, IV	39		43					401	69	86.8
			Trapé *et al*, 2003	IIIA, IIIB, IV						10	17	21		100
			Wang *et al*, 2011	I, II, III, IV	1		5		64			38		94.4
	Prediction	4	Pang *et al*, 2013	I, II, IIIA, IIIB, IV	34		44			27	28	143		71.7
			Wang *et al*, 2010	I, II, III, IV	5		14		66			42		85.0
			Nisman *et al*, 2008	IIIA, IIIB, IV						5	18	37		100
CYFRA 21-1			Jin *et al*, 2010	IIIB, IV							39	72		100
			Merle *et al*, 2004	IIIA, IIIB						18	26			100
	Treatment monitoring	7	Ardizzoni *et al*, 2006	III, IV					41			76		100
			Yang *et al*, 2012	IIIB, IV					53			45		100
			Wang *et al*, 2011	I, II, III, IV	1		5		64			38		94.4
			Pang *et al*, 2013	I, II, IIIA, IIIB, IV	34		44			27	28	143		71.7

**Marker**	**Clinical question**	**Studies,** ***n***	**Study**	**Stages**	**Tumour response criteria**	**Time of evaluation**
			Trapé *et al*, 2003	IIIA, IIIB, IV	WHO	Unknown
			Wang *et al*, 2011	I, II, III, IV	WHO	6–8 weeks post-tx
	Prediction	5	Pang *et al*, 2013	I, II, IIIA, IIIB, IV	RECIST	2 months post-tx
			Liu *et al*, 2014	I, II, III, IV	RECIST	After second cycle
			Wang *et al*, 2010	I, II, III, IV	WHO	2 months post-tx
			Salgia *et al*, 2001	III/IIIB, IV^b^	Unknown	Unknown
			Jin *et al*, 2010	IIIB, IV	RECIST	After second cycle
			Ardizzoni *et al*, 2006	III, IV	WHO	After second cycle
	Treatment monitoring	8	Yang *et al*, 2012	IIIB, IV	WHO, RECIST	After second cycle
CEA			Arrieta *et al*, 2013	III, IV	RECIST	After second cycle
			Ishiguro *et al*, 2010	IB, IIB, IIIA, IIIB	RECIST	Post-chemo, presurgery
			Pang *et al*, 2013	I, II, IIIA, IIIB, IV	RECIST	2 months post-tx
			Liu *et al*, 2014	I, II, III, IV	RECIST	After second cycle
			Trapé *et al*, 2003	IIIA, IIIB, IV	WHO	Unknown
			Wang *et al*, 2011	I, II, III, IV	WHO	6–8 weeks post-tx
	Prediction	4	Pang *et al*, 2013	I, II, IIIA, IIIB, IV	RECIST	2 months post-tx
			Wang *et al*, 2010	I, II, III, IV	WHO	2 months post-tx
			Nisman *et al*, 2008	IIIA, IIIB, IV	Unknown	After second cycle
CYFRA 21-1			Jin *et al*, 2010	IIIB, IV	RECIST	After second cycle
			Merle *et al*, 2004	IIIA, IIIB	WHO	After second cycle
	Treatment monitoring	7	Ardizzoni *et al*, 2006	III, IV	WHO	After second cycle
			Yang *et al*, 2012	IIIB, IV	WHO, RECIST	After second cycle
			Wang *et al*, 2011	I, II, III, IV	WHO	6–8 weeks post-tx
			Pang *et al*, 2013	I, II, IIIA, IIIB, IV	RECIST	2 months post-tx

Abbreviations: AUC=area under the curve; CEA=carcinoembryonic antigen; chemo=chemotherapy; CR=complete response; CYFRA 21-1=cytokeratin-19 fragments; PD=progressive disease; PR=partial response; RECIST=Response Evaluation Criteria in Solid Tumors; SD=stable disease; SN=sensitivity; SP=specificity; tx=treatment; WHO=World Health Organization.

a The first marker measurement was taken pretreatment in all of the studies.

b The information ‘late stage (unresectable and metastatic disease)' is given.

c Information as given in the publication. Owing to dropouts, the number of patients considered in the analysis may be lower.

d Separate results for late-stage patients available.

**Table 2:** Summary of available studies for the comparison (PD) *vs* (CR+PR+SD)

**Marker**	**Clinical question**	**Studies,** ***n***	**Study**	**Stages**	**Second marker measurement**^a^	**Responders/non-responders,** ***n***	**SN/SP available**	**AUC available**
	Prediction	2	Trapé *et al*, 2003	IIIA, IIIB, IV	—	32/16	Yes	No
			Wang *et al*, 2010	I, II, III, IV	—	79/48	Yes	No
CEA	Treatment	2	Jin *et al*, 2010	IIIB, IV	After second cycle	93/18	Yes	No
	monitoring		Arrieta *et al*, 2013	III, IV	After second cycle	140/40	Yes	Yes
	Prediction	3	Holdenrieder *et al*, 2006	IIIA, IIIB, IV	—	219/92	Yes	No
			Trapé *et al*, 2003	IIIA, IIIB, IV	—	32/16	Yes	No
CYFRA 21-1			Wang *et al*, 2010	I, II, III, IV	—	79/48	Yes	Yes
	Treatment	2 (3^b^)	Jin *et al*, 2010	IIIB, IV	After second cycle	27/84	Yes	No
	monitoring		Nisman *et al*, 2008	IIIA, IIIB, IV	After second cycle	10/35	Yes	No
			Holdenrieder *et al*, 2006	IIIA, IIIB, IV	After day 8 of first cycle	219/92	Yes	No

					**Patients with corresponding stage,** ***n***^c^								
**Marker**	**Clinical question**	**Studies,** ***n***	**Study**	**Stages**	**I**	**IB**	**II**	**IIB**	**III**	**IIIA**	**IIIB**	**IV**	**Patients with stage III–IV, %**
	Prediction	2	Trapé *et al*, 2003	IIIA, IIIB, IV						10	17	21	100
CEA			Wang *et al*, 2010	I, II, III, IV	5		14		66			42	85.0
	Treatment monitoring	2	Jin *et al*, 2010	IIIB, IV							39	72	100
			Arrieta *et al*, 2013	III, IV					28			152	100
	Prediction	3	Holdenrieder *et al*, 2006	IIIA, IIIB, IV						13	100	198	100
			Trapé *et al*, 2003	IIIA, IIIB, IV						10	17	21	100
CYFRA 21-1			Wang *et al*, 2010	I, II, III, IV	5		14		66			42	85.0
	Treatment monitoring	2	Jin *et al*, 2010	IIIB, IV							39	72	100
			Nisman *et al*, 2008	IIIA, IIIB, IV						5	18	37	100

**Marker**	**Clinical question**	**Studies,** ***n***	**Study**	**Stages**	**Tumour response criteria**	**Time of evaluation**
	Prediction	2	Trapé *et al*, 2003	IIIA, IIIB, IV	WHO	Unknown
CEA			Wang *et al*, 2010	I, II, III, IV	WHO	2 months post-tx
	Treatment monitoring	2	Jin *et al*, 2010	IIIB, IV	RECIST	After second cycle
			Arrieta *et al*, 2013	III, IV	RECIST	After second cycle
	Prediction	3	Holdenrieder *et al*, 2006	IIIA, IIIB, IV	WHO	After second cycle
			Trapé *et al*, 2003	IIIA, IIIB, IV	WHO	Unknown
CYFRA 21-1			Wang *et al*, 2010	I, II, III, IV	WHO	2 months post-tx
	Treatment monitoring	2	Jin *et al*, 2010	IIIB, IV	RECIST	After second cycle
			Nisman *et al*, 2008	IIIA, IIIB, IV	Unknown	After second cycle

Abbreviations: AUC=area under the curve; CEA=carcinoembryonic antigen; CR=complete response; CYFRA 21-1=cytokeratin-19 fragments; PD=progressive disease; PR=partial response; RECIST=Response Evaluation Criteria in Solid Tumors; SD=stable disease; SN=sensitivity; SP=specificity; tx=treatment; WHO=World Health Organization.

a The first marker measurement was taken pretreatment in all of the studies.

b For consistency in the timing of the second marker measurement, results of the study by Holdenrieder *et al* (2006) for CYFRA 21-1 treatment monitoring were excluded from further analysis.

c Information as given in the publication. Owing to dropouts, the number of patients considered in the analysis may be lower.

**Table 3:** Results of meta-analysis for AUC and sensitivity/specificity

**Comparison**	**Marker**	**Clinical question**	**Studies,** ***n***	**AUC** (**95% CI**)	**Tau**^**2**^	***Q***	***I***^**2**^
(CR+PR) *vs* (SD+PD)	CEA	Treatment monitoring	5	0.728 (0.599–0.871)	1.683	35.774	88.384
	CYFRA 21-1		5	0.724 (0.667–0.785)	0.070	4.805	22.738
**Comparison**	**Marker**	**Clinical question**	**Studies,** ***n***	**Sensitivity (95% CI)**	**Specificity (95% CI)**	**Tau**^**2**^ **sensitivity**	**Tau**^**2**^ **specificity**
(CR+PR) *vs* (SD+PD)	CEA	Prediction	5	0.568 (0.495–0.638)	0.536 (0.417–0.652)	0.053	0.233
		Treatment monitoring	8	0.747 (0.642–0.830)	0.698 (0.594–0.785)	0.349	0.345
	CYFRA 21-1	Prediction	4	0.505 (0.377–0.633)	0.672 (0.582–0.751)	0.210	0.076
		Treatment monitoring	7	0.791 (0.715–0.851)	0.606 (0.538–0.671)	0.139	0.059
(PD) *vs* (CR+PR+SD)	CEA	Prediction	2	0.625 (0.501–0.734)	0.522 (0.430–0.614)	0.000	0.000
		Treatment monitoring	2	0.817 (0.661–0.911)	0.317 (0.066–0.755)	0.113	1.812
	CYFRA 21-1	Prediction	3	0.593 (0.289–0.839)	0.660 (0.292–0.902)	1.166	1.806
		Treatment monitoring	2	0.844 (0.638–0.943)	0.714 (0.383–0.909)	0.244	0.859

Abbreviations: AUC=area under the curve; CEA=carcinoembryonic antigen; CI=confidence interval; CR=complete response; CYFRA 21-1=cytokeratin-19 fragments; PD=progressive disease; PR=partial response; SD=stable disease.

## References

[bib1] Ardizzoni A, Cafferata MA, Tiseo M, Filiberti R, Marroni P, Grossi F, Paganuzzi M (2006) Decline in serum carcinoembryonic antigen and cytokeratin 19 fragment during chemotherapy predicts objective response and survival in patients with advanced nonsmall cell lung cancer. Cancer 107: 2842–2849. 17103443 10.1002/cncr.22330

[bib2] Arrieta O, Villarreal-Garza C, Martínez-Barrera L, Morales M, Dorantes-Gallareta Y, Peña-Curiel O, Contreras-Reyes S, Macedo-Pérez EO, Alatorre-Alexander J (2013) Usefulness of serum carcinoembryonic antigen (CEA) in evaluating response to chemotherapy in patients with advanced non small-cell lung cancer: a prospective cohort study. BMC Cancer 13: 254. 23697613 10.1186/1471-2407-13-254 PMC3665670

[bib3] Barak V, Holdenrieder S, Nisman B, Stieber P (2010) Relevance of circulating biomarkers for the therapy monitoring and follow-up investigations in patients with non-small cell lung cancer. Cancer Biomark 6: 191–196. 20660964 10.3233/CBM-2009-0129 PMC12922862

[bib4] Egger M, Davey Smith G, Schneider M, Minder C (1997) Bias in meta-analysis detected by a simple, graphical test. BMJ 315: 629–634. 9310563 10.1136/bmj.315.7109.629 PMC2127453

[bib5] Ganti AK, Mulshine JL (2006) Lung cancer screening. Oncologist 11: 481–487. 16720848 10.1634/theoncologist.11-5-481

[bib6] Glas AS, Lijmer JG, Prins MH, Bonsel GJ, Bossuyt PM (2003) The diagnostic odds ratio: a single indicator of test performance. J Clin Epidemiol 56: 1129–1135. 14615004 10.1016/s0895-4356(03)00177-x

[bib7] Gridelli C, Rossi A, Maione P (2003) Treatment of non-small-cell lung cancer: state of the art and development of new biologic agents. Oncogene 22: 6629–6638. 14528288 10.1038/sj.onc.1206957

[bib8] Hanley JA, McNeil BJ (1982) The meaning and use of the area under a receiver operating characteristic (ROC) curve. Radiology 143: 29–36. 7063747 10.1148/radiology.143.1.7063747

[bib9] Holdenrieder S, Nagel D, Stieber P (2010) Estimation of prognosis by circulating biomarkers in patients with non-small cell lung cancer. Cancer Biomark 6: 179–190. 20660963 10.3233/CBM-2009-0128 PMC12922861

[bib10] Holdenrieder S, Stieber P (2010) Circulating apoptotic markers in the management of non-small cell lung cancer. Cancer Biomark 6: 197–210. 20660965 10.3233/CBM-2009-0130 PMC12922863

[bib11] Holdenrieder S, Stieber P, von Pawel J, Raith H, Nagel D, Feldmann K, Seidel D (2004) Circulating nucleosomes predict the response to chemotherapy in patients with advanced non-small cell lung cancer. Clin Cancer Res 10: 5981–5987. 15447981 10.1158/1078-0432.CCR-04-0625

[bib12] Holdenrieder S, Stieber P, von Pawel J, Raith H, Nagel D, Feldmann K, Seidel D (2006) Early and specific prediction of the therapeutic efficacy in non-small cell lung cancer patients by nucleosomal DNA and cytokeratin-19 fragments. Ann NY Acad Sci 1075: 244–257. 17108218 10.1196/annals.1368.033

[bib13] Holdenrieder S, von Pawel J, Dankelmann E, Duell T, Faderl B, Markus A, Siakavara M, Wagner H, Feldmann K, Hoffmann H, Raith H, Nagel D, Stieber P (2008) Nucleosomes, ProGRP, NSE, CYFRA 21-1, and CEA in monitoring first-line chemotherapy of small cell lung cancer. Clin Cancer Res 14: 7813–7821. 19047109 10.1158/1078-0432.CCR-08-0678

[bib14] Ishiguro F, Fukui T, Mori S, Katayama T, Sakakura N, Hatooka S, Mitsudomi T (2010) Serum carcinoembryonic antigen level as a surrogate marker for the evaluation of tumor response to chemotherapy in nonsmall cell lung cancer. Ann Thorac Cardiovasc Surg 16: 242–247. 21057440

[bib15] Jin B, Huang AM, Zhong RB, Han BH (2010) The value of tumor markers in evaluating chemotherapy response and prognosis in Chinese patients with advanced non-small cell lung cancer. Chemotherapy 56: 417–423. 21079400 10.1159/000317580

[bib16] Liu H, Gu X, Lv T, Wu Y, Xiao Y, Yuan D, Li Y, Song Y (2014) The role of serum carcinoembryonic antigen in predicting responses to chemotherapy and survival in patients with non-small cell lung cancer. J Cancer Res Ther 10: 239–243. 25022372 10.4103/0973-1482.136541

[bib17] Mahadevia PJ, Fleisher LA, Frick KD, Eng J, Goodman SN, Powe NR (2003) Lung cancer screening with helical computed tomography in older adult smokers: a decision and cost-effectiveness analysis. JAMA 289: 313–322. 12525232 10.1001/jama.289.3.313

[bib18] Merle P, Janicot H, Filaire M, Roux D, Bailly C, Vincent C, Gachon F, Tchirkov A, Kwiatkowski F, Naam A, Escande G, Caillaud D, Verrelle P (2004) Early CYFRA 21-1 variation predicts tumor response to chemotherapy and survival in locally advanced non-small cell lung cancer patients. Int J Biol Markers 19: 310–315. 15646838 10.1177/172460080401900409

[bib19] Molina R, Filella X, Augé JM, Fuentes R, Bover I, Rifa J, Moreno V, Canals E, Viñolas N, Marquez A, Barreiro E, Borras J, Viladiu P (2003) Tumor markers (CEA, CA 125, CYFRA 21–1, SCC and NSE) in patients with non-small cell lung cancer as an aid in histological diagnosis and prognosis. Comparison with the main clinical and pathological prognostic factors. Tumour Biol 24: 209–218. 14654716 10.1159/000074432

[bib20] Molina R, Holdenrieder S, Auge JM, Schalhorn A, Hatz R, Stieber P (2010) Diagnostic relevance of circulating biomarkers in patients with lung cancer. Cancer Biomark 6: 163–178. 20660962 10.3233/CBM-2009-0127 PMC12922860

[bib21] Molina R, Marrades RM, Auge JM, Escudero JM, Viñolas N, Reguart N, Ramirez J, Filella X, Molins L, Agustí A (2016) Assessment of a combined panel of six serum tumor markers for lung cancer. Am J Respir Crit Care Med 193: 427–437. 26465739 10.1164/rccm.201404-0603OC

[bib22] National Comprehensive Cancer Network (2016) NCCN clinical practice guidelines in oncology. Non-small cell lung cancer, version 3.2017—16 November 2016. Available at https://www.nccn.org accessed 09 December 2016.

[bib23] Nisman B, Biran H, Heching N, Barak V, Ramu N, Nemirovsky I, Peretz T (2008) Prognostic role of serum cytokeratin 19 fragments in advanced non-small-cell lung cancer: association of marker changes after two chemotherapy cycles with different measures of clinical response and survival. Br J Cancer 98: 77–79. 18087272 10.1038/sj.bjc.6604157 PMC2359680

[bib24] Pang L, Wang J, Jiang Y, Chen L (2013) Decreased levels of serum cytokeratin 19 fragment CYFRA 21-1 predict objective response to chemotherapy in patients with non-small cell lung cancer. Exp Ther Med 6: 355–360. 24137188 10.3892/etm.2013.1171 PMC3786728

[bib25] Reitsma JB, Glas AS, Rutjes AW, Scholten RJ, Bossuyt PM, Zwinderman AH (2005) Bivariate analysis of sensitivity and specificity produces informative summary measures in diagnostic reviews. J Clin Epidemiol 58: 982–990. 16168343 10.1016/j.jclinepi.2005.02.022

[bib26] Rutter CM, Gatsonis CA (2001) A hierarchical regression approach to meta-analysis of diagnostic test accuracy evaluations. Stat Med 20: 2865–2884. 11568945 10.1002/sim.942

[bib27] Salgia R, Harpole D, Herndon JE 2nd, Pisick E, Elias A, Skarin AT (2001) Role of serum tumor markers CA 125 and CEA in non-small cell lung cancer. Anticancer Res 21: 1241–1246. 11396194

[bib28] Schalhorn A, Fuerst H, Stieber P (2001) Tumor markers in lung cancer. J Lab Med 25: 353–361.

[bib29] The R Foundation (2015) The R project for statistical computing Available at http://www.r-project.org/ accessed 7 July 2016.

[bib30] Trapé J, Buxo J, Pérez de Olaguer J, Vidal C (2003) Tumor markers as prognostic factors in treated non-small cell lung cancer. Anticancer Res 23: 4277–4281. 14666638

[bib31] Uhlig S, Hettwer K, Colson B, Baldauf H (2015) Carcinoembryonic antigen (CEA) and cytokeratin 19 fragment (CYFRA 21-1) for assessment of therapy response in advanced non-small cell lung cancer: a systematic review and meta-analysis. PROSPERO 2015:CRD42015029974. Available at: http://www.crd.york.ac.uk/PROSPERO/display_record.asp?ID=CRD42015029974 (accessed 7 July 2016.

[bib32] Vollmer RT, Govindan R, Graziano SL, Gamble G, Garst J, Kelley MJ, Christenson RH (2003) Serum CYFRA 21–1 in advanced stage non-small cell lung cancer: an early measure of response. Clin Cancer Res 9: 1728–1733. 12738727

[bib33] Walter SD (2002) Properties of the summary receiver operating characteristic (SROC) curve for diagnostic test data. Stat Med 21: 1237–1256. 12111876 10.1002/sim.1099

[bib34] Wang J, Yi Y, Li B, Wang Z, Sun H, Zhang P, Huang W (2010) CYFRA21-1 can predict the sensitivity to chemoradiotherapy of non-small-cell lung carcinoma. Biomarkers 15: 594–601. 20649505 10.3109/1354750X.2010.504308

[bib35] Wang J, Zhang N, Li B, Wang Z, Sun H, Yi Y, Huang W (2011) Decline of serum CYFRA 21-1 during chemoradiotherapy of NSCLC: a probable predictive factor for tumor response. Tumour Biol 32: 689–695. 21409421 10.1007/s13277-011-0169-2

[bib36] Whiting PF, Rutjes AWS, Westwood ME, Mallett S, Deeks JJ, Reitsma JB, Leeflang MM, Sterne JA, Bossuyt PM QUADAS-2 Group (2011) QUADAS-2: a revised tool for the quality assessment of diagnostic accuracy studies. Ann Intern Med 155: 529–536. 22007046 10.7326/0003-4819-155-8-201110180-00009

[bib37] Yang L, Chen X, Li Y, Yang J, Tang L (2012) Declines in serum 21-1 and carcinoembryonic antigen as predictors of chemotherapy response and survival in patients with advanced non-small cell lung cancer. Exp Ther Med 4: 243–248. 22970029 10.3892/etm.2012.570 PMC3439001

[bib38] Zhi XY, Yu JM, Shi YK (2015) Chinese guidelines on the diagnosis and treatment of primary lung cancer (2015 version). Cancer 121(Suppl 17): 3165–3181. 26331823 10.1002/cncr.29550

