# Supplementary material for: Carcinoembryonic antigen and cytokeratin-19 fragments for assessment of therapy response in non-small cell lung cancer: a systematic review and meta-analysis
Source: Br J Cancer. 2017 Mar 9;116(8):1037–45. doi: 10.1038/bjc.2017.45 (PMC5396105; doi:10.1038/bjc.2017.45)
Supplement: Supplementary Material [file bjc201745x1.docx]

**Supplementary material**

Supplementary Figure 1. Funnel plots for AUC for CEA and CYFRA 21-1 for the comparison [CR+PR] versus [SD+PD]. The white region corresponds to *P* values > 0.10, the grey-shaded region corresponds to *P* values between 0.10 and 0.05, and the dark grey-shaded area corresponds to *P* values between 0.05 and 0.01.

AUC = area under the curve; CEA = carcinoembryonic antigen; CR = complete response; CYFRA 21-1 = cytokeratin-19 fragments; PD = progressive disease; PR = partial response; SD = stable disease.

Supplementary Figure 2. Funnel plots for In DOR for CEA and CYFRA 21-1 for the comparison [CR+PR] versus [SD+PD]. The white region corresponds to *P* values > 0.1, the grey-shaded region corresponds to *P* values between 0.10 and 0.05, and the dark grey-shaded area corresponds to *P* values between 0.05 and 0.01.

CEA = carcinoembryonic antigen; CR = complete response; CYFRA 21-1 = cytokeratin-19 fragments; DOR = diagnostic odds ratio; In = logarithmic; PD = progressive disease; PR = partial response; SD = stable disease.

Supplementary Figure 3. Study-specific cut-off values and sensitivity/specificity for (A) CEA, and (B) CYFRA 21-1, for the comparison [CR+PR] versus [SD+PD]**.** For the clinical question ‘Treatment Monitoring’, a higher x-axis value indicates a greater reduction in marker level, thus values above the cut-off are indicative of response.

CEA = carcinoembryonic antigen; CR = complete response; CYFRA 21-1 = cytokeratin-19 fragments; PD = progressive disease; PR = partial response; SD = stable disease.

Supplementary Figure 4. Forest plots for In DOR for the comparison [CR+PR] versus [SD+PD].

CEA = carcinoembryonic antigen; CR = complete response; CYFRA 21-1 = cytokeratin-19 fragments; DOR = diagnostic odds ratio; In = logarithmic; PD = progressive disease; PR = partial response; SD = stable disease.

**Supplementary Table 1 Studies excluded from the meta-analysis with reasons**

| **Study** | **Reasons for non-eligibility** |
| --- | --- |
| Karnak *et al*, 2001 | Compares a control group with lung cancer patients |
| Massacesi *et al*, 2003 | Analyses CEA and NSE in combination |
| Holdenrieder *et al*, 2004 | Contingency data are not fully available |
| Okamoto *et al*, 2005 | Involves patients who received one or more prior therapies |
| Chiu *et al*, 2007 | Involves patients who received one or more prior therapies |
| Hotta *et al*, 2007 | Involves patients who received one or more prior therapies |
| Jung *et al*, 2011 | Involves patients who received one or more prior therapies |
| Jung *et al*, 2012 | Involves patients who received one or more prior therapies |
| Alm El-Din *et al*, 2012 | The absolute CYFRA 21-1 level after the second chemotherapy cycle rather than the pre-treatment level was used for response assessment |
| Qin *et al*, 2013 | Contingency data are not fully available |
| Facchinetti *et al*, 2015 | Involves patients who received one or more prior therapies |

Abbreviations: CEA = carcinoembryonic antigen; CYFRA 21-1 = cytokeratin-19 fragments; NSE = neuron-specific enolase.

Non-eligible studies:

Karnak D, Ulubay G, Kayacan O, Beder S, Ibis E, Oflaz G (2001) Evaluation of CYFRA 21-1: a potential tumor marker for non-small cell lung carcinomas. *Lung* **179:** 57–65.

Massacesi C, Rocchi MB, Marcucci F, Pilone A, Galeazzi M, Bonsignori M (2003) Serum tumor markers may precede instrumental response to chemotherapy in patients with metastatic cancer. *Int J Biol Markers* **18:** 295–300.

Holdenrieder S, Stieber P, von Pawel J, Raith H, Nagel D, Feldmann K, Seidel D (2004) Circulating nucleosomes predict the response to chemotherapy in patients with advanced non-small cell lung cancer. *Clin Cancer Res* **10(18 Pt 1):** 5981–5987.

Okamoto T, Nakamura T, Ikeda J, Maruyama R, Shoji F, Miyake T, Wataya H, Ichinose Y (2005) Serum carcinoembryonic antigen as a predictive marker for sensitivity to gefitinib in advanced non-small cell lung cancer. *Eur J Cancer* **41:** 1286–1290.

Chiu CH, Shih YN, Tsai CM, Liou JL, Chen YM, Perng RP (2007) Serum tumor markers as predictors for survival in advanced non-small cell lung cancer patients treated with gefitinib. *Lung Cancer* **57:** 213–221.

Hotta K, Kiura K, Tabata M, Takigawa N, Fujiwara Y, Umemura S, Tanimoto M (2007) Role of early serial change in serum carcinoembryonic antigen levels as a predictive marker for radiological response to gefitinib in Japanese patients with non-small cell lung cancer. *Anticancer Res* **27:** 1737–1741.

Jung M, Kim SH, Lee YJ, Hong S, Kang YA, Kim SK, Chang J, Rha SY, Kim JH, Kim DJ, Cho BC (2011) Prognostic and predictive value of CEA and CYFRA 21-1 levels in advanced non-small cell lung cancer patients treated with gefitinib or erlotinib. *Exp Ther Med* **2:** 685–693.

Jung M, Kim SH, Lee YJ, Hong S, Kang YA, Kim SK, Chang J, Rha SY, Kim JH, Kim DJ, Cho BC (2012) Prognostic and predictive value of carcinoembryonic antigen and cytokeratin-19 fragments levels in advanced non-small cell lung cancer patients treated with gefitinib or erlotinib. *Yonsei Med J* **53:** 931–939.

Alm El-Din MA, Farouk G, Nagy H, Abd Elzaher A, Abo El-Magd GH (2012) Cytokeratin-19 fragments, nucleosomes and neuron-specific enolase as early measures of chemotherapy response in non-small cell lung cancer. *Int J Biol Markers* **27:** e139–146.

Qin HF, Qu LL, Liu H, Wang SS, Gao HJ (2013) Serum CEA level change and its significance before and after gefitinib therapy on patients with advanced non-small cell lung cancer. *Asian Pac J Cancer Prev* **14:** 4205–4208.

Facchinetti F, Aldigeri R, Aloe R, Bortesi B, Ardizzoni A, Tiseo M (2015) CEA serum level as early predictive marker of outcome during EGFR-TKI therapy in advanced NSCLC patients. *Tumour Biol* **36:** 5943–5951.

**Supplementary Table 2 Cut-off values for CEA and CYFRA 21-1, chemotherapy and criteria for tumour response for eligible studies**

| Study | Cut-off for response assessment^a^ | | | | Chemotherapy | Treatment | Tumour response evaluation criteria | Tumour response examination method |
| --- | --- | --- | --- | --- | --- | --- | --- | --- |
|  | CEA | | CYFRA 21-1 | |  |  |  |  |
|  | Prediction | Treatment  monitoring | Prediction | Treatment  monitoring |  |  |  |  |
| Salgia *et al*, 2001 | Unknown |  |  |  | Unknown | Chemo | Unknown | Radiological |
| Trapé *et al*, 2003 | 5.0 ng/mL,  *5.0 ng/mL* |  | 3.6 ng/mL,  *3.6 ng/mL* |  | Platinum-based chemo; CBDCA/PTX;  CDDP/PTX;  CDDP/VP16;  CDDP/VP16/DOC;  CDDP/G | Chemo + partial radio | WHO | Imaging |
| Merle *et al*, 2004 |  |  |  | 65% decrease | Platinum-based chemo;  CDDP/VNR/IFOS;  CDDP/VP16/IFOS; | Chemo + radio | WHO | CT |
| Ardizzoni *et al*, 2006 |  | 20% decrease |  | 20% decrease | 2nd- or 3rd-generation, platinum-based chemo;  non-platinum-based chemo | Chemo | WHO | CT |
| Holdenrieder *et al*, 2006 |  |  | *14.0 ng/mL* |  | Platinum-based chemo;  CBDCA/MC/VB;  CBDCA/MC/VNR;  CDDP/G | Chemo | WHO |  |
| Nisman *et al*, 2008 |  |  |  | 62% decrease^b^,  *35% decrease^b^* | Mainly platinum-based chemo;  CDDP/G;  CBDCA/PTX;  CBDCA/VP16;  others | Chemo | Unknown | CT |
| Wang *et al*, 2010 | 5.03 ng/mL,  *3.4 ng/mL* |  | 4.12 ng/mL,  *3.3 ng/mL* |  | Platinum-based chemo + radio;  CDDP/VNR/G | Chemo + radio | WHO | CT or PET/CT |
| Ishiguro *et al*, 2010 |  | 60% decrease^c^ |  |  | Platinum-based chemo;  CBDCA/PTX;  CBDCA/DOC;  CBDCA/VNR;  CBDCA/VP-16;  CDDP/VP-16;  CDDP/MC/VD;  DOC | Chemo + adjuvant surgery | RECIST | CT |
| Jin *et al*, 2010 |  | Baseline,  *Baseline* |  | Baseline,  *Baseline* | Platinum-based chemo;  CDDP/VNR;  CDDP/G;  CDDP/PTX;  CDDP/DOC;  CDDP/VNR/IFOS | Chemo | RECIST | CT |
| Wang *et al*, 2011 | 3.4 ng/mL |  | 3.3 ng/mL | 18.48% decrease | Platinum-based chemo + radio;  CDDP/VNR | Chemo + radio | WHO | CT or PET/CT |
| Yang *et al*, 2012 |  | 25% decrease^d^ |  | 60% decrease^e^ | Platinum-based chemo | Chemo | WHO, RECIST | CT |
| Arrieta *et al*, 2013 |  | 14% decrease^f^,  *18% increase^f^* |  |  | Platinum-based chemo | Chemo or TKI | RECIST | CT |
| Pang *et a*l, 2013 | 5.0 ng/mL | Unknown | 3.3 ng/mL | Unknown | 3rd generation platinum-based chemo + PTX or DOC, G, P | Chemo or TKI, surgery | RECIST | CT |
| Liu *et al*, 2014 | 9.7 ng/mL | 5.28% decrease |  |  | Standardised combination chemotherapy | Chemo | RECIST | CT |

^a^ Cut-offs for the comparison [CR+PR] versus [SD+PD] in non-italics; Cut-offs for the comparison [PD] versus [CR+PR+SD] in italics.

^b^ Pre- or post-level > 3.2 ng/mL.

^c^ Pre-level > 5 ng/mL.

^d^ Pre-level of either CEA or CYFRA 21-1 > 3.4 ng/mL.

^e^ Pre-level of either CEA or CYFRA 21-1 > 3.2 ng/mL.

^f^ Pre-level > 10 ng/mL.

Abbreviations: CBDCA = carboplatin; CDDP = cisplatin; CEA = carcinoembryonic antigen; Chemo = chemotherapy; CR = complete response; CT = computed tomography; CYFRA 21-1 = cytokeratin-19 fragments; DOC = docetaxel; G = gemcitabine; IFOS = ifosfamide; MC = mytomycin C; P = pemetrexed; PD = progressive disease; PET = positron emission tomography; PR = partial response; PTX = paclitaxel; radio = radiotherapy; RECIST = Response Evaluation Criteria in Solid Tumors; SD = stable disease; TKI = tyrosine kinase inhibitor; VB = vinblastin; VD = vindesin; VNR = vinorelbine; VP-16 = etoposide; WHO = World Health Organization.

It is important to note that, in the Arrieta 2013 study, the comparison [PD] versus [CR+PR+SD] is considered as such. Accordingly, the cut-off is given as an 18% increase, meaning that an *increase* of at least 18% is required for the change in marker level to be taken as an indication of progression. In the Nisman 2008 study, the opposite comparison is considered (i.e. non-progression vs. progression). For this reason, the cut-off is given as a 35% reduction, meaning that a *decrease* of at least 35% is required for the change in marker level to be taken as indicative of non-progression. In the Jin 2010, Trapé 2003, and Wang 2010 studies, the comparison [PD] versus [CR+PR+SD] is not considered and the cut-offs given here were intended for the [CR+PR] versus [SD+PD] comparison.

**Supplementary Table 3 Studies that analysed both markers for each question, or analysed both questions for each marker**

|  | Clinical question | |
| --- | --- | --- |
|  | Prediction | Treatment monitoring |
| CEA and CYFRA 21-1 | Trapé *et al*, 2003; Wang *et al*, 2010; Wang *et al*, 2011;  Pang *et al*, 2013 | Ardizzoni *et al*, 2006; Jin *et al*, 2010; Yang *et al*, 2012;  Pang *et al*, 2013 |
| CEA | Pang *et al*, 2013; Liu *et al*, 2014 | |
| CYFRA 21-1 | Wang *et al*, 2011; Pang *et al*, 2013 | |

Abbreviations: CEA = carcinoembryonic antigen; CYFRA 21-1 = cytokeratin-19 fragments.

**Supplementary Table 4 Contingency table for the two clinical questions**

|  | Response to therapy | |
| --- | --- | --- |
| Clinical question | Responder  [CR+PR] | Non-responder  [SD+PD] |
| Prediction:   - Pre-treatment marker level *below* cut-off   Treatment monitoring:   - Strong reduction in marker level (≥ cut-off) | True positive | False positive |
| Prediction:   - Pre-treatment marker level *above* cut-off   Treatment monitoring:   - Weak reduction in marker level (< cut-off) | False negative | True negative |
|  | Responder  [CR+PR+SD] | Non-responder  [PD] |
| Prediction:   - Pre-treatment marker level *above* cut-off   Treatment monitoring:   - Weak decrease in marker level (< cut-off) | False positive | True positive |
| Prediction:   - Pre-treatment marker level *below* cut-off   Treatment monitoring:   - Strong decrease in marker level (≥ cut-off) | True negative | False negative |

Abbreviations: CR = complete response; PD = progressive disease; PR = partial response; SD = stable disease.

**Supplementary Table 5 Results of meta-analysis for DOR**

| Comparison | Marker | Clinical question | Studies, *n* | DOR [95% CI] | In DOR [95% CI] | Tau^2^ | Q | I^2^ |
| --- | --- | --- | --- | --- | --- | --- | --- | --- |
| [CR+PR] versus [SD+PD] | CEA | Prediction | 5 | 1.49 [1.03–2.16] | 0.40 [0.03–0.76] | 0.05 | 3.78 | 0.00 |
|  |  | Treatment monitoring | 8 | 6.89 [3.40–13.95] | 1.93 [1.23–2.62] | 0.73 | 8.29 | 15.59 |
|  | CYFRA 21-1 | Prediction | 4 | 2.16 [1.49–3.13] | 0.77 [0.41–1.14] | 0.00 | 1.63 | 0.00 |
|  |  | Treatment monitoring | 7 | 6.42 [3.50–11.79] | 1.86 [1.25–2.47] | 0.38 | 5.29 | 0.00 |
| [PD] versus [CR+PR+SD] | CEA | Prediction | 2 | 1.82 [0.97–3.41] | 0.60 [–0.03–1.22] | 0.00 | 0.10 | 0.00 |
|  |  | Treatment monitoring | 2 | 1.97 [0.48–8.09] | 0.68 [–0.73–2.10] | 0.74 | 1.00 | 0.00 |
|  | CYFRA 21-1 | Prediction | 3 | 3.16 [2.01–4.96] | 1.15 [0.70–1.61] | 0.00 | 1.00 | 0.00 |
|  |  | Treatment monitoring | 2 | 14.73 [5.01–43.29] | 2.69 [1.61–3.77] | 0.00 | 0.36 | 0.00 |

Abbreviations: CEA = carcinoembryonic antigen; CI = confidence interval; CR = complete response; CYFRA 21-1 = cytokeratin-19 fragments; DOR = diagnostic odds ratio; In = logarithmic; PD = progressive disease; PR = partial response; SD = stable disease.

**Supplementary Table 6 Summary of available studies for the comparison [CR+PR] versus [SD+PD] with information relating to ethnic group and assay type**

| Marker | Clinical question | Studies, *n* | Study | Stages | Ethnic group | Assay type | Cut-off | SN^a^ | SP | AUC |
| --- | --- | --- | --- | --- | --- | --- | --- | --- | --- | --- |
| CEA | Prediction | 5 | Trapé *et al*, 2003 | III–IV | Caucasian (Spain) | Automated | 5.0 ng/ml | 0.583 | 0.583 |  |
|  |  |  | Wang *et al*, 2011 | Mixed | Asian (China) | Automated | 3.4 ng/ml | 0.439 | 0.725 |  |
|  |  |  | Pang *et al*, 2013 | Mixed | Asian (China) | Automated | 5.0 ng/ml | 0.573 | 0.506 |  |
|  |  |  | Liu *et al*, 2014 | Mixed | Asian (China) | Automated | 9.7 ng/ml | 0.590 | 0.392 |  |
|  |  |  | Wang *et al*, 2010 | Mixed | Asian (China) | Automated | 5.03 ng/ml | 0.671 | 0.542 | 0.563 |
|  | Treatment monitoring | 8 | Salgia *et al*, 2001 | III–IV | Other (USA) | Unknown | Unknown | 0.818 | 0.731 |  |
|  |  |  | Jin *et al*, 2010 | III–IV | Asian (China) | Manual | Baseline | 0.741 | 0.583 |  |
|  |  |  | Ardizzoni *et al*, 2006 | III–IV | Caucasian (Italy) | Automated | 20% decrease | 0.553 | 0.750 | 0.650 |
|  |  |  | Yang *et al*, 2012 | III–IV | Asian (China) | Manual | 25% decrease | 0.711 | 0.679 | 0.629 |
|  |  |  | Arrieta *et al*, 2013 | III–IV | Other (Mexico) | Automated | 14% decrease | 0.902 | 0.899 | 0.945 |
|  |  |  | Ishiguro *et al*, 2010 | Mixed | Asian (Japan) | Automated | 60% decrease | 0.818 | 0.692 |  |
|  |  |  | Pang *et al*, 2013 | Mixed | Asian (China) | Automated | Unknown | 0.818 | 0.553 | 0.738 |
|  |  |  | Liu *et al*, 2014 | Mixed | Asian (China) | Automated | 5.28% decrease | 0.610 | 0.625 | 0.615 |
| CYFRA 21-1 | Prediction | 4 | Trapé *et al*, 2003 | III–IV | Caucasian (Spain) | Automated | 3.6 ng/ml | 0.375 | 0.708 |  |
|  |  |  | Wang *et al*, 2011 | Mixed | Asian (China) | Automated | 3.3 ng/ml | 0.421 | 0.765 |  |
|  |  |  | Pang *et al*, 2013 | Mixed | Asian (China) | Automated | 3.3 ng/ml | 0.515 | 0.640 |  |
|  |  |  | Wang *et al*, 2010 | Mixed | Asian (China) | Automated | 4.12 ng/ml | 0.671 | 0.604 | 0.616 |
|  | Treatment monitoring | 7 | Nisman *et al*, 2008 | III–IV | Other (Israel) | Manual | 62% decrease | 0.900 | 0.600 |  |
|  |  |  | Jin *et al*, 2010 | III–IV | Asian (China) | Manual | Baseline | 0.667 | 0.560 |  |
|  |  |  | Merle *et al*, 2004 | III–IV | Caucasian (France) | Automated | 65% decrease | 0.769 | 0.778 | 0.778 |
|  |  |  | Ardizzoni *et al*, 2006 | III–IV | Caucasian (Italy) | Automated | 20% decrease | 0.809 | 0.550 | 0.690 |
|  |  |  | Yang *et al*, 2012 | III–IV | Asian (China) | Manual | 60% decrease | 0.778 | 0.774 | 0.727 |
|  |  |  | Wang *et al*, 2011 | Mixed | Asian (China) | Automated | 18.48% decrease | 0.719 | 0.510 | 0.626 |
|  |  |  | Pang *et al*, 2013 | Mixed | Asian (China) | Automated | Unknown | 0.897 | 0.607 | 0.779 |

Abbreviations: AUC = area under the curve; CEA = carcinoembryonic antigen; CR = complete response; CYFRA 21-1 = cytokeratin-19 fragments; PD = progressive disease; PR = partial response; SD = stable disease; SN = sensitivity; SP = specificity.

^a^ Sensitivity/specificity values calculated on the basis of contingency tables.

**Supplementary Table 7 Summary of available studies for the comparison [PD] versus [CR+PR+SD] with information relating to ethnic group and assay type**

| Marker | Clinical question | Studies, *n* | Study | Stages | Ethnic group | Assay type | Cut-off | SN^a^ | SP | AUC |
| --- | --- | --- | --- | --- | --- | --- | --- | --- | --- | --- |
| CEA | Prediction | 2 | Trapé *et al*, 2003 | III–IV | Caucasian (Spain) | Automated | 5.0 ng/ml | 0.625 | 0.563 |  |
|  |  |  | Wang *et al*, 2010 | Mixed | Asian (China) | Automated | 3.4 ng/ml | 0.625 | 0.506 |  |
|  | Treatment monitoring | 2 | Jin *et al*, 2010 | III–IV | Asian (China) | Manual | Baseline | 0.778 | 0.548 |  |
|  |  |  | Arrieta *et al*, 2013 | III–IV | Other (Mexico) | Automated | 18% increase | 0.850 | 0.150 | 0.911 |
| CYFRA 21-1 | Prediction | 3 | Holdenrieder *et al*, 2006 | III–IV | Caucasian (Germany) | Automated | 14.0 ng/ml | 0.304 | 0.900 |  |
|  |  |  | Trapé *et al*, 2003 | III–IV | Caucasian (Spain) | Automated | 3.6 ng/ml | 0.813 | 0.406 |  |
|  |  |  | Wang *et al*, 2010 | Mixed | Asian (China) | Automated | 3.3 ng/ml | 0.667 | 0.544 | 0.616 |
|  | Treatment monitoring | 2 | Jin *et al*, 2010 | III–IV | Asian (China) | Manual | Baseline | 0.889 | 0.570 |  |
|  |  |  | Nisman *et al*, 2008 | III–IV | Other (Israel) | Manual | 35% decrease | 0.789 | 0.846 |  |

Abbreviations: AUC = area under the curve; CEA = carcinoembryonic antigen; CR = complete response; CYFRA 21-1 = cytokeratin-19 fragments; PD = progressive disease; PR = partial response; SD = stable disease; SN = sensitivity; SP = specificity.

It is important to note that, in the Arrieta *et al* 2013 study, the comparison [PD] versus [CR+PR+SD] is considered as such. Accordingly, the cut-off is given as an 18% increase, meaning that an *increase* of at least 18% is required for the change in marker level to be taken as an indication of progression. In the Nisman *et al* 2008 study, the opposite comparison is considered (i.e., non-progression vs. progression). For this reason, the cut-off is given as a 35% reduction, meaning that a *decrease* of at least 35% is required for the change in marker level to be taken as indicative of non-progression. In the Jin *et al* 2010, Trapé *et al* 2003, and Wang *et al* 2010 studies, the comparison [PD] versus [CR+PR+SD] is not considered and the cut-offs given here were intended for the [CR+PR] versus [SD+PD] comparison.

^a^ Sensitivity/specificity values calculated on the basis of contingency tables.
