# Supplementary figures and images for: Carcinoembryonic antigen and cytokeratin-19 fragments for assessment of therapy response in non-small cell lung cancer: a systematic review and meta-analysis
Source: Br J Cancer. 2017 Mar 9;116(8):1037–45. doi: 10.1038/bjc.2017.45 (PMC5396105; doi:10.1038/bjc.2017.45)

# [CR+PR] versus [SD+PD]

**CEA**

change (5 studies, AUC 0.83,  $P=0.038$ )

**CYFRA 21-1**

change (5 studies, AUC 0.72,  $P=0.847$ )

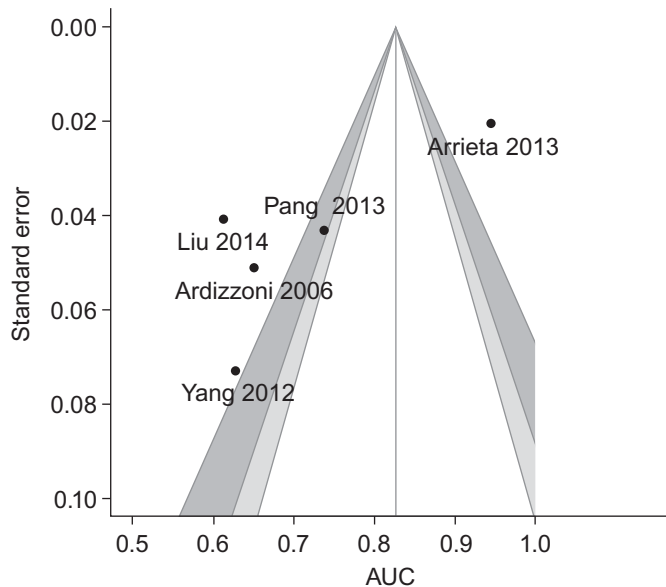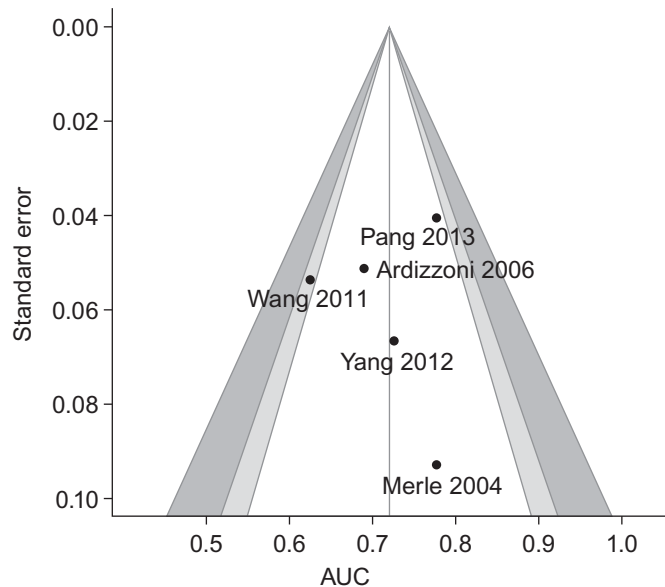

Supplement: Supplementary Figure 1 [file bjc201745x2.pdf]

CYFRA 21-1, change

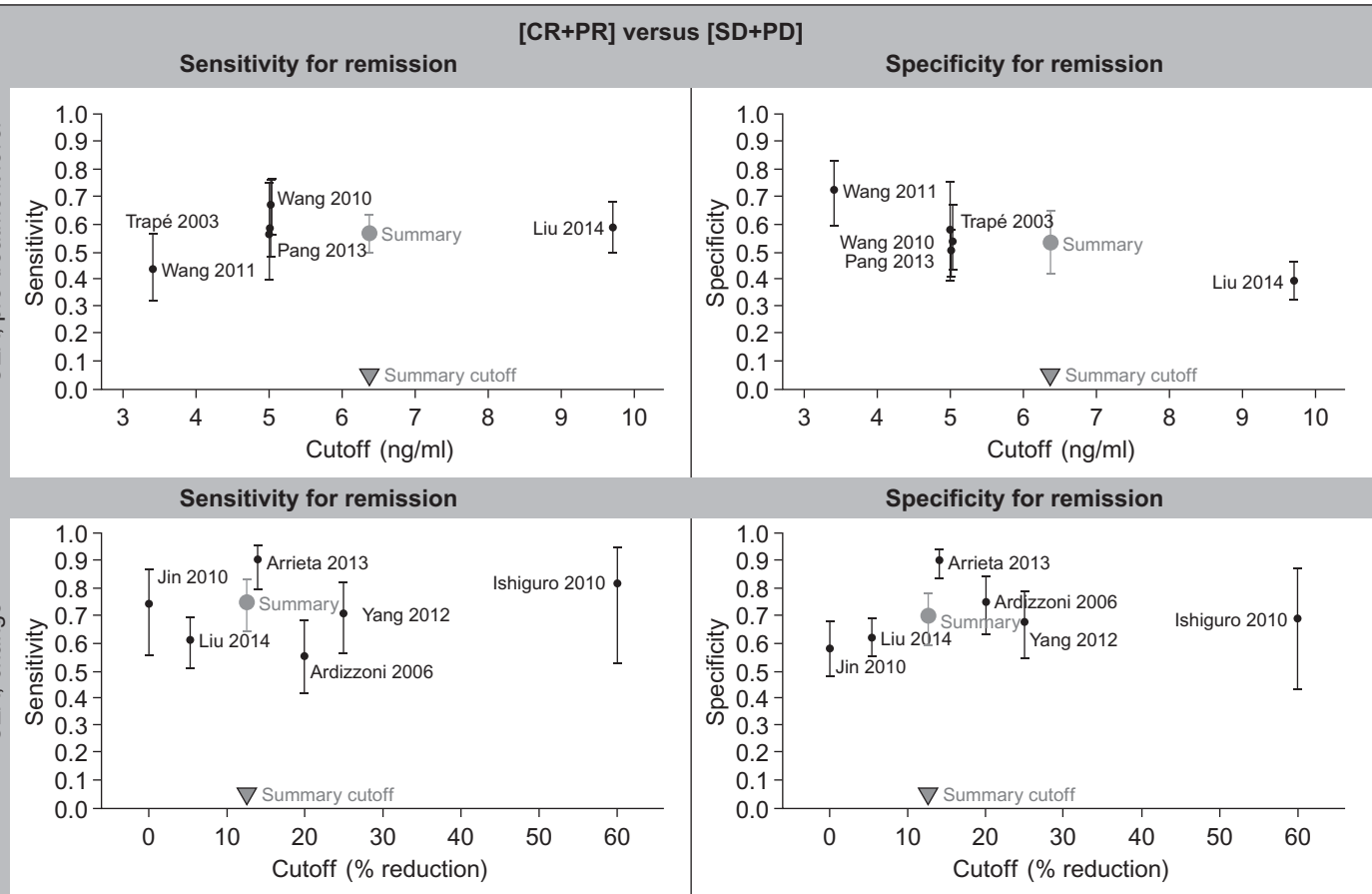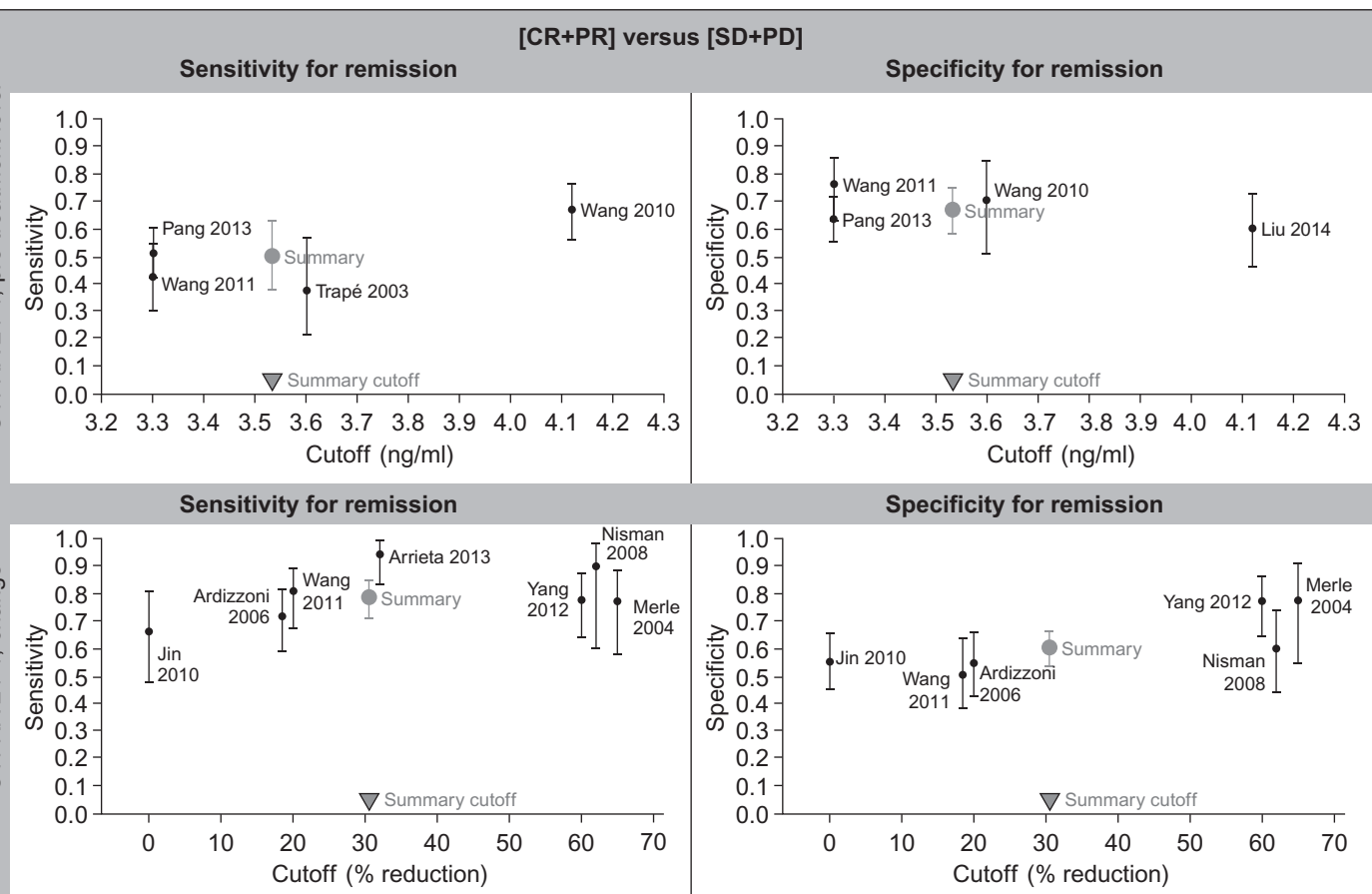

Supplement: Supplementary Figure 3 [file bjc201745x4.pdf]

[CR+PR] versus [SD+PD]  
CEA, pre-treatment level

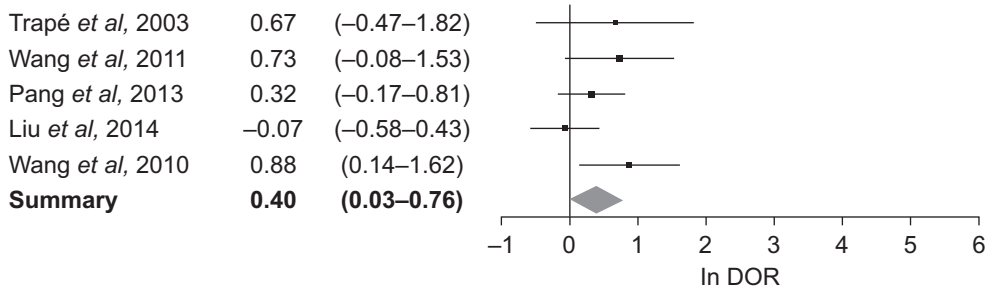

CEA, change

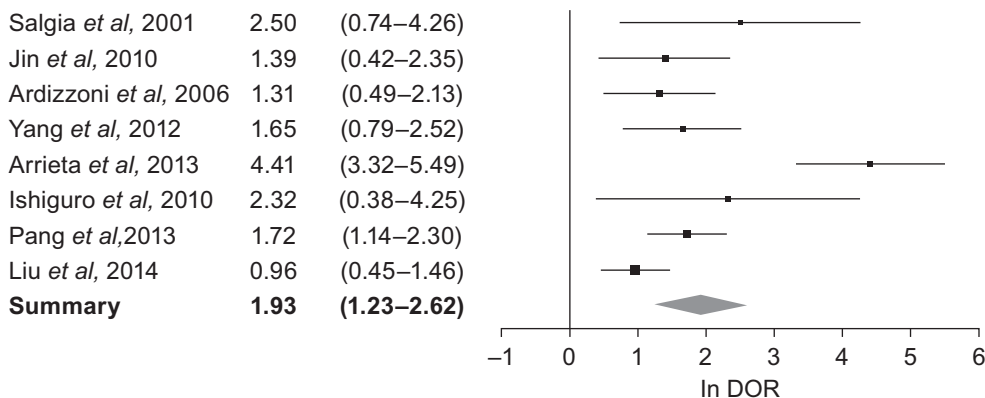

CYFRA 21-1, pre-treatment level

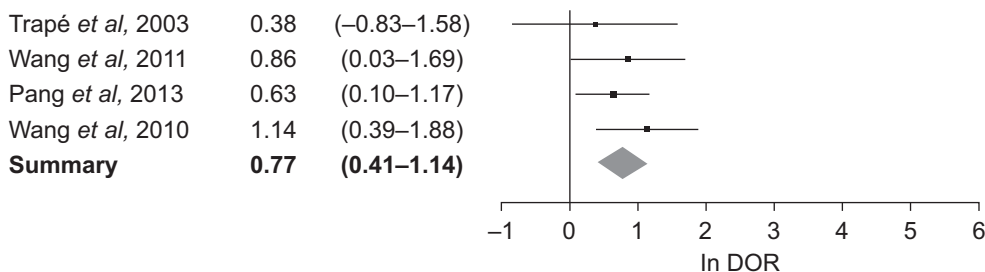

CYFRA 21-1, change

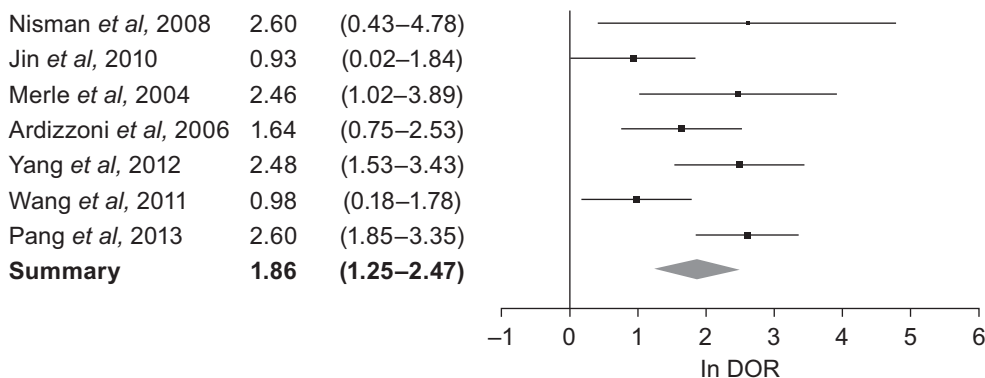

Supplement: Supplementary Figure 4 [file bjc201745x5.pdf]
