# Supplementary material for: Carcinoembryonic antigen and cytokeratin-19 fragments for assessment of therapy response in non-small cell lung cancer: a systematic review and meta-analysis
Source: Br J Cancer. 2017 Mar 9;116(8):1037–45. doi: 10.1038/bjc.2017.45 (PMC5396105; doi:10.1038/bjc.2017.45)
Supplement: Supplementary Figure 2 [file bjc201745x3.pdf]

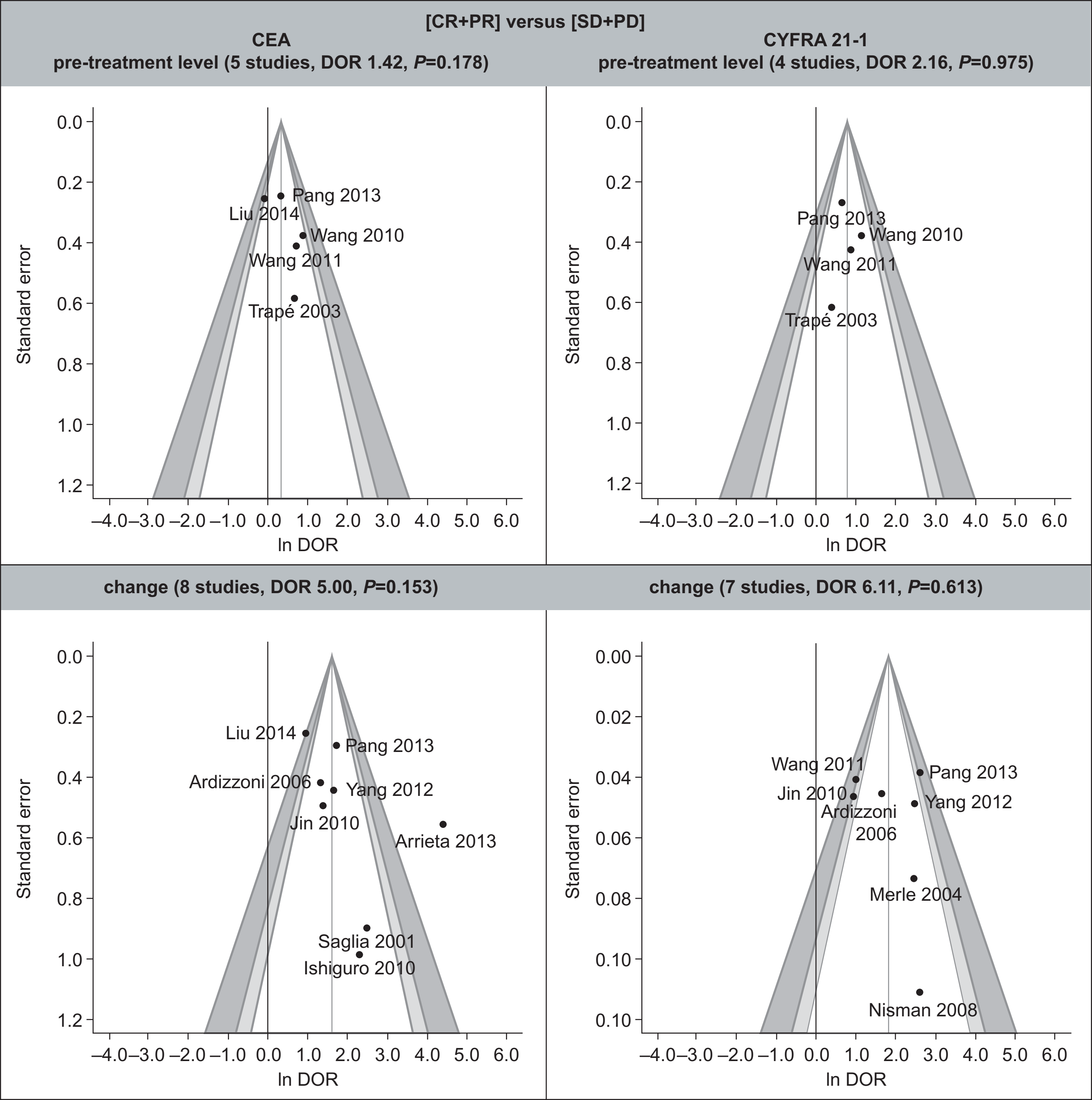

[CR+PR] versus [SD+PD]
CEA
pre-treatment level (5 studies, DOR 1.42, P=0.178)
CYFRA 21-1
pre-treatment level (4 studies, DOR 2.16, P=0.975)
Standard error
ln DOR
Pang 2013
Liu 2014
Wang 2010
Wang 2011
Trapé 2003
change (8 studies, DOR 5.00, P=0.153)
change (7 studies, DOR 6.11, P=0.613)
Ardizzoni 2006
Yang 2012
Jin 2010
Arrieta 2013
Saglia 2001
Ishiguro 2010
Ardizzoni
2006
Merle 2004
Nisman 2008
